# Gas Sensors Based on Chemically Reduced Holey Graphene Oxide Thin Films

**DOI:** 10.1186/s11671-019-3060-5

**Published:** 2019-07-01

**Authors:** Ming Yang, Yanyan Wang, Lei Dong, Zhiyong Xu, Yanhua Liu, Nantao Hu, Eric Siu-Wai Kong, Jiang Zhao, Changsi Peng

**Affiliations:** 1 School of Optoelectronic Science and Engineering & Collaborative Innovation Center of Suzhou Nano Science and Technology, Soochow University, Suzhou, 215006 People’s Republic of China; 2 Key Lab of Advanced Optical Manufacturing Technologies of Jiangsu Province & Key Lab of Modern Optical Technologies of Education Ministry of China, Soochow University, Suzhou, 215006 People’s Republic of China; 3 Jiangsu Provincial Engineering Laboratory for RF Integration and Micropackaging, College of Electronic and Optical Engineering & College of Microelectronics, Nanjing University of Posts and Telecommunications, Nanjing, 210023 People’s Republic of China; 4 Key Laboratory for Thin Film and Microfabrication of Ministry of Education, Department of Micro/Nano Electronics, School of Electronic Information and Electrical Engineering, Shanghai Jiao Tong University, Shanghai, 200240 People’s Republic of China

**Keywords:** Graphene oxide, Reduced graphene oxide, Holey graphene, NH_3_ gas sensor

## Abstract

The nanosheet stacking phenomenon in graphene thin films significantly deteriorates their gas-sensing performance. This nanosheet stacking issue should be solved and reduced to enhance the gas detection sensitivity. In this study, we report a novel ammonia (NH_3_) gas sensor based on holey graphene thin films. The precursors, holey graphene oxide (HGO) nanosheets, were prepared by etching graphene under UV irradiation with Fenton reagent (Fe^2+^/Fe^3+^/H_2_O_2_). Holey graphene was prepared by the reduction of HGO (rHGO) with pyrrole. Holey graphene thin-film gas sensors were prepared by depositing rHGO suspensions onto the electrodes. The resulting sensing devices show excellent response, sensitivity, and selectivity to NH_3_. The resistance change is 2.81% when the NH_3_ level is as low as 1 ppm, whereas the resistance change is 11.32% when the NH_3_ level is increased to 50 ppm. Furthermore, the rHGO thin-film gas sensor could be quickly restored to their initial states without the stimulation with an IR lamp. In addition, the devices showed excellent repeatability. The resulting rHGO thin-film gas sensor has a great potential for applications in numerous sensing fields because of its low cost, low energy consumption, and outstanding sensing performance.

**Electronic supplementary material:**

The online version of this article (10.1186/s11671-019-3060-5) contains supplementary material, which is available to authorized users.

## Introduction

Chemiresistive sensors play more and more important roles in domains such as environmental monitoring, industrial production, medicine, military, and public safety [1–6]. Today, solid-state gas sensors still suffer from issues related to long-term stability and accuracy of detection [7]. Nanomaterials such as nanowires, carbon nanotubes, and graphene [8–10] have shown great potential in the next generation of gas sensors due to their high aspect ratio, large specific surface area, excellent electronic properties, and simple fabrication [11–13].

Graphene, a single-layer structure of carbon atoms in a two-dimensional (2D) honeycomb lattice, has been widely reported as an excellent sensing material, owing to its high specific surface area, unique electrical properties, and excellent mechanical, chemical, and thermal properties [14–19]. Its electronic properties strongly depend on surface adsorption, which can change the density of carriers. Graphene and reduced graphene oxide (rGO) show excellent sensing performance towards numerous gases including NO_2_, NH_3_, CO, ethanol, H_2_O, trimethylamine, HCN, and dimethyl methylphosphonate [13, 20–28]. The rGO obtained by the chemical reduction of graphene oxide (GO) has great potential application in chemiresistors owing to its cost-effectiveness, large-scale production, and large usable surface areas [29–32]. Most previous studies focused on 2D structures [33–38]. However, 2D graphene sheets can be assembled into three-dimensional (3D) foamed graphene network or nanoporous structure to increase the surface area [39–43]. Although rGO has outstanding potential as a gas sensor with miniature, low-cost, and portable characteristics, it is still not widely used, thus slowing down the commercial application of rGO-based sensing devices.

Two main methods have been reported for fabricating chemiresistive sensors based on nanomaterials: (1) Electrodes are deposited on the top of sensing materials [44]. This constitutes a complex process, and exquisite skills are required. (2) An rGO dispersion is drop-casted onto a surface containing the electrodes [45]. It is difficult to perfect dispersion-casting techniques to ensure the reproducibility of sensing devices. Hence, it is desirable to fabricate porous graphene thin-film gas-sensing devices with characteristic facile drop-casting techniques.

In this study, we report a novel NH_3_ sensor based on holey graphene thin films. Holey graphene oxide (HGO) obtained by the etching of GO by photo-Fenton reaction [46] was used as a precursor to assemble thin films. Reduced holey graphene oxide (rHGO) was formed by the reduction of HGO with pyrrole. rHGO thin-film gas sensors were prepared by dropping rHGO suspensions onto the electrodes. The performance of gas sensor prepared by this method is significantly better than that of rGO device based on the dispersion method. Easy, green, and reproducible sensors can be prepared based on rHGO films. These sensors have excellent performance, low-cost, miniature, and portable characteristics. As a result, a new avenue is prepared for the application of rHGO thin films in the gas-sensing field.

## Materials and Methods

### Material

The natural graphite powder used in this study was purchased from Tianyuan, Shandong, China. Pyrrole was obtained from Suzhou Chemical Reagents (China) and purified by distillation. Ferrous sulfate (FeSO_4_) was purchased from Shanghai Chemical Reagents, China. All other chemicals were purchased from Suzhou Chemical Reagents, China, and used as received without further purification. All the organic solvents were purified by distillation.

### Preparation of HGO

GO was synthesized using the improved Hummers method [31]. Briefly, 57.5 mL of H_2_SO_4_ was added to a glass flask containing graphite (2 g). After stirring for 30 min, 1 g of NaNO_3_ was added, and the mixture was stirred for 2 h in an ice bath. The flask was transferred to a 35 °C water bath, and 7.3 g KMnO_4_ was added. The mixture was stirred for 3 h. Then, 150 mL pure water was added, and the reaction was continued for another 30 min. Then, 55 mL of 4% H_2_O_2_ was added, and the solution was stirred for 30 min to obtain a GO suspension. The resulting GO suspension was rinsed with a large amount of aqueous HCl (3%) three times. The product obtained after washing with water was dried at 40 °C in a vacuum oven for 24 h. The GO aqueous dispersion at a concentration of 0.5 mg/mL was sonicated and stored for later use.

Twenty milliliters H_2_O_2_ and 100 μL FeSO_4_ were added to the GO dispersion (5 mL); then, the mixture was continued to sonicate for 10 min. The pH of the mixture was adjusted to 4 by adding aqueous HCl (1%). Subsequently, the photo-Fenton reaction of GO was carried out in the mixture dispersion [46]. After several minutes, some small holes appeared on the surface of GO. The reaction was dialyzed in deionized water for 1 week to remove the metal ions, unreacted H_2_O_2_, and other small molecular species produced by the reaction.

### Preparation of rHGO

The rHGO was obtained by reducing HGO with pyrrole. First, 50 mL of HGO (1 mg/mL) was obtained by ultrasonication at room temperature for 1 h, and pyrrole (1 mg) dispersed in ethanol (10 mL) was added. The mixture was further sonicated for 20 min and stirred under reflux in an oil bath at 95 °C for 12 h. Finally, the mixture was filtered using a G5 sintered glass and rinsed with DMF and ethanol. Thus, rHGO was prepared.

### Fabrication of Gas Sensor Based on rHGO

The electrodes for rHGO sensors were fabricated using a conventional microfabrication process, as reported in our previous studies [45, 47, 48]. The interdigitated arrays of electrodes (8 pairs) possess a finger length of 600 μm and a gap size of 5 μm. The electrodes were prepared by sputtering Cr (10 nm) and Au (180 nm) on a lithographic pattern. The photoresist was then removed by the lift-off process. Finally, the electrodes were sonicated in acetone, rinsed with a large amount of deionized water, and then purged with nitrogen for later use.

rHGO sensors were prepared as follows: 0.05 μL of rHGO ethanol suspension (1 mg/mL) was dropped onto the electrode using a syringe. After the electrodes were dried in air, a conductive network structure was formed on the surface of electrode.

### Gas-Sensing Measurement

**Fig. 1:**
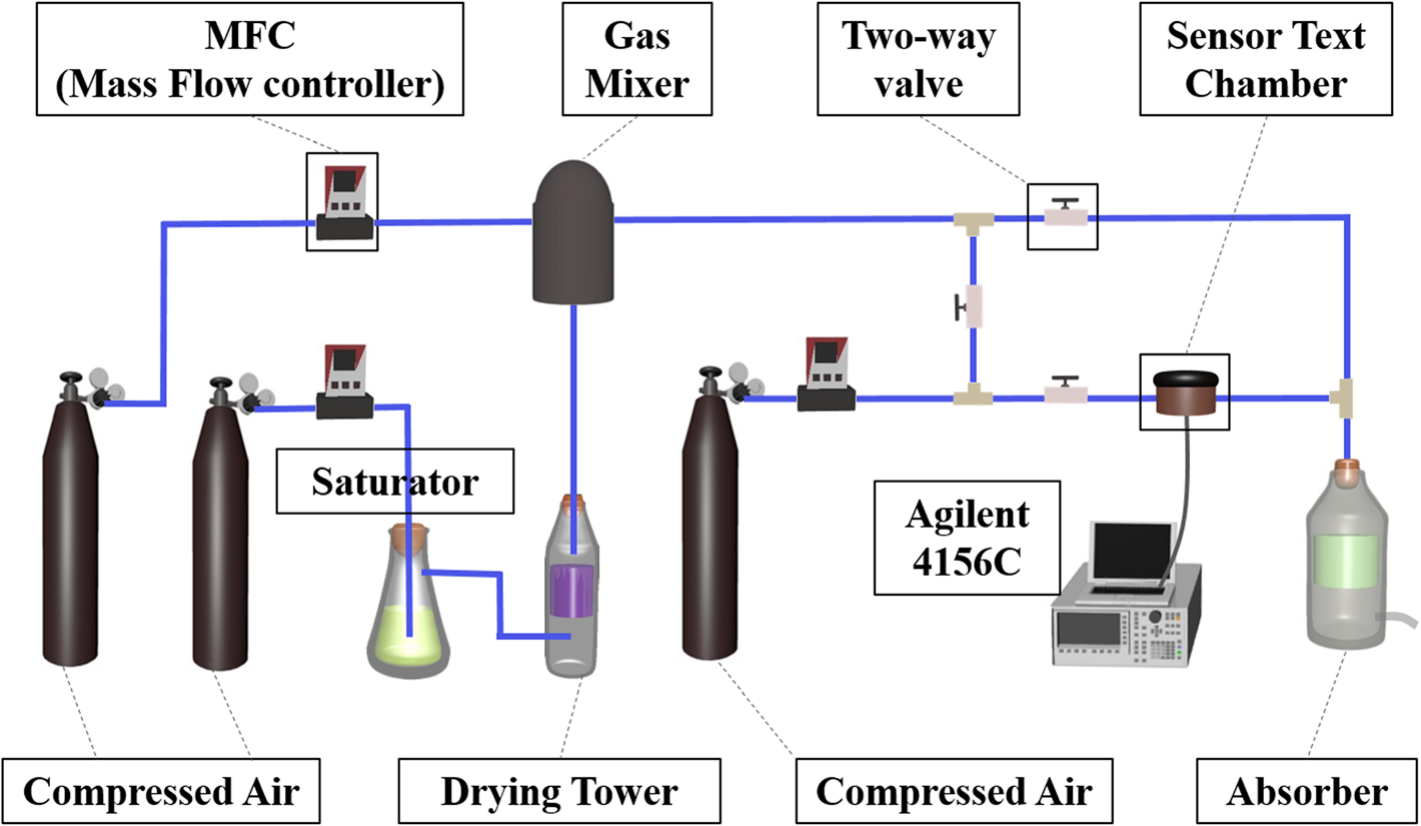
Schematic diagram of experimental setup for gas-sensing test

The sensing properties of rHGO sensors were evaluated using a self-made sensor system, as shown in Fig. 1. Dry NH_3_ was bubbled by blowing dry air into 4% NH_3_ aqueous solution, subsequently through a drying tube with NaOH flakes. The concentration of NH_3_ can be controlled by air dilution and monitored using a mass flow meter. The flow rate of balance gas (dry air) was controlled at 1.0 L/min. All the sensing measurements were carried out using a precision semiconductor tester (Agilent 4156C) at room temperature (25 °C). The response of sensor was measured by the resistance change at a voltage of 500 mV.

### Characterization

AFM measurement was conducted using a Dimension Icon instrument (Veeco, Plainview, NY, USA). XPS measurements were performed using a Thermo Scientific Escalab 250 X-ray photoelectron spectrometer (Thermo Fisher Scientific Inc., UK) using monochromated Al K_α_ X-ray beams as the excitation source (1486.6 eV). Raman scattering was carried out using a Jobin-Yvon HR-800 Raman spectrometer equipped with a 633-nm laser source. The morphologies of samples were observed using a scanning electron microscope (Hitachi S-4800).

## Results and Discussion

### Synthesis and Characterization of HGO and rHGO

**Fig. 2:**
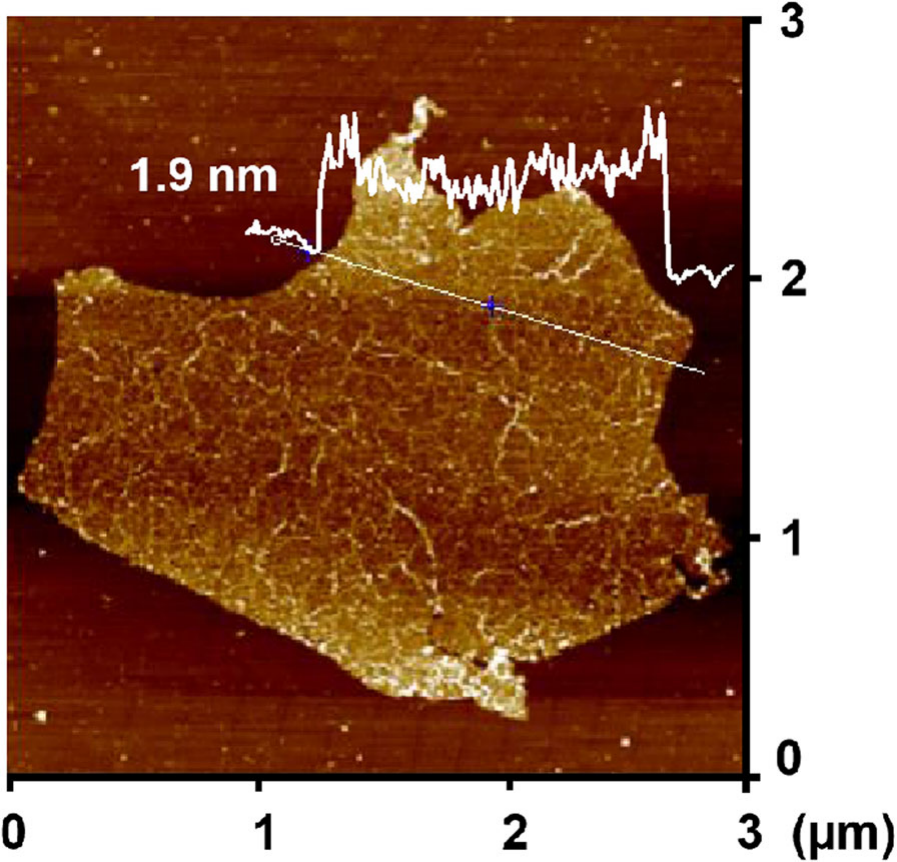
AFM image of GO sheets after reaction with Fenton reagent under UV irradiation for 1 h

An improved Hummers method was used to oxidize the graphite, thus forming a stable aqueous dispersion of GO. The photo-Fenton reaction of GO was induced at the junction of carbon and oxygen atoms, cleaving the C–C bonds [46]. The progress of photo-Fenton reaction of GO was measured by atomic force microscopy (AFM). As shown in Fig. 2 and Additional file 1: Figure S1, after 1 h of reaction, many small holes are observed on the surface of GO sheets. It can be seen from Fig. 2 and Additional file 1: Figure S2 that the thickness of graphene before etching is about 1 nm, and the thickness of graphene after etching is about 1.9 nm. The results indicate that a single layer of graphene was prepared [49]. As a result, HGO sheets well dispersed in water were obtained, and the sheet layer maintained a large-dimensional characteristic.

**Fig. 3:**
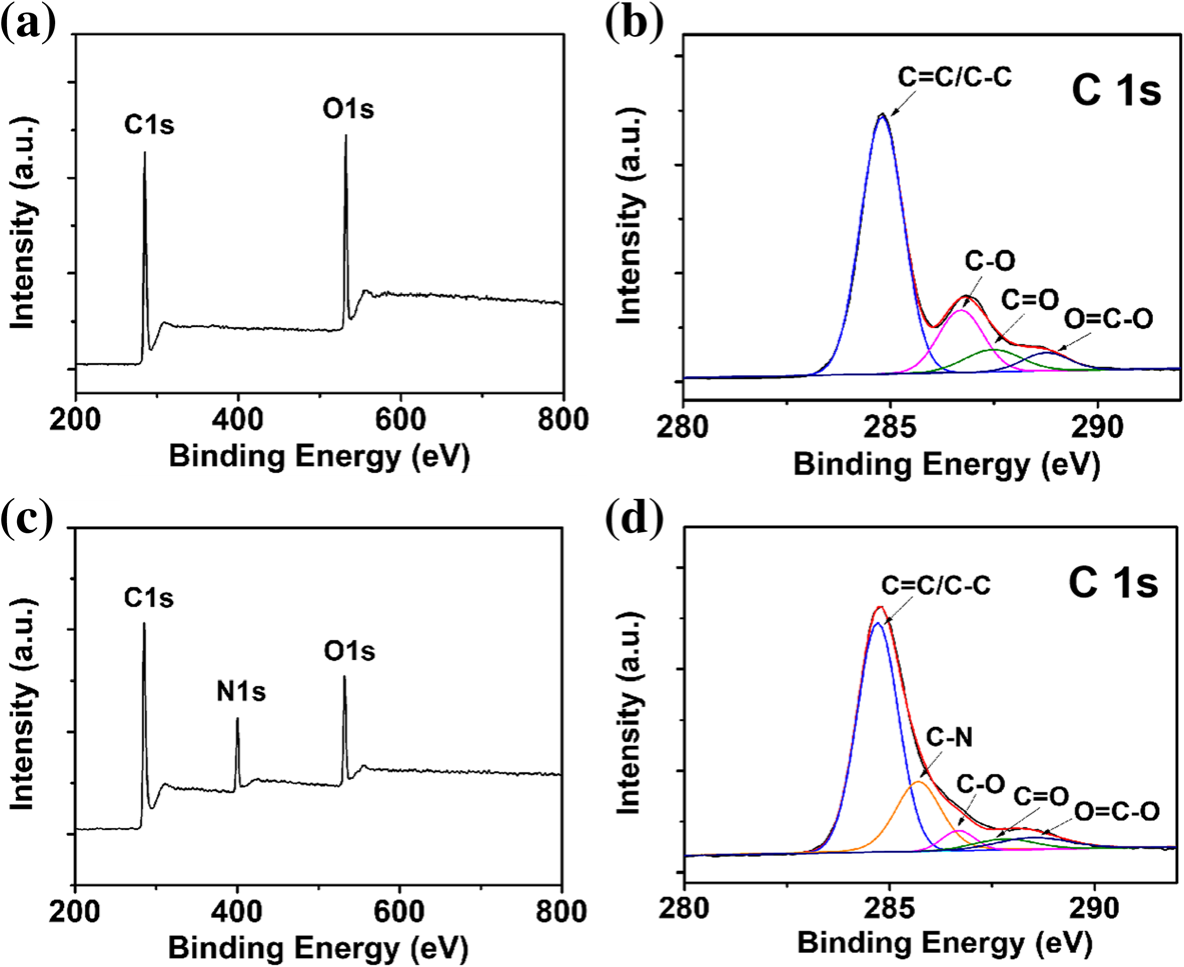
XPS spectra of Cls of HGO before (**a**) and after the reduction (**b**). XPS spectra of HGO (**c**) and rHGO (**d**)

X-ray photoelectron spectroscopy (XPS) also provided evidence for the reduction of HGO to rHGO during the hydrothermal process. Figure 3b and d show the XPS spectra of C1s of HGO and rHGO. In the XPS C1s spectra of HGO (Fig. 3b), four typical peaks at 284.8, 286.7, 287.5, and 288.7 eV are assigned to C–C/C=C, C–O, C=O, and O–C=O groups, respectively [50]. As the reduction reaction occurs, the peak intensities of C–O and C=O groups in the Cls spectra of XPS are significantly reduced in rHGO. Moreover, the scanning curve in Fig. 3a, c shows that a new peak of N1s appears in the scanning curve of rHGO relative to the scanning curve of HGO, suggesting polypyrrole (PPy) molecules had been attached on the surface of rGO after reduction [51, 52]. The ratio of C/O of HGO and rHGO were found to be 2.2 and 5.1, respectively. The increased C/O ratio in rHGO indicated that most of the oxygen-containing functional groups were removed from HGO during reduction by pyrrole.

**Fig. 4:**
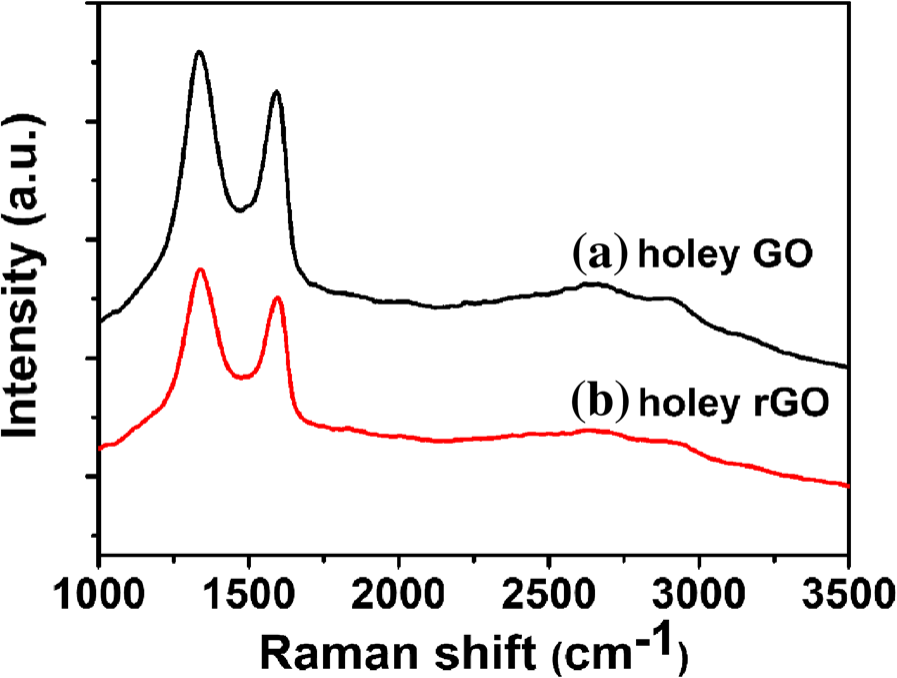
Raman spectra of **a** HGO and **b** rHGO with an excitation wavelength of 632 nm

Raman spectroscopy is a commonly used tool to measure the order of crystal structure of carbon atoms. The presence of D band at 1346 cm^−1^ and G band at 1597 cm^−1^ is demonstrated by the Raman spectrum as shown in Fig. 4. Currently, the D band represents the degree of disorder of graphene crystal structure due to the destruction of C=C bond between the edge and oxygen-containing functional group, and the G band can be attributed to the mutual stretching of sp^2^ hybrid atom pair in graphite lattice, namely the hexagonal closeness of graphene carbon atom [53]. The relative intensity ratio of I_D_/I_G_ reflects the change in surface functional groups before and after reduction. The reduction has also been verified by the decrease of FWHM of the D peak as shown in Fig. 4b [54]. After the reduction with pyrrole, the calculated I_D_/I_G_ ratio decreased from 1.29 (HGO) to 1.12 (rHGO). This is because of the increase in average size of crystalline sp^2^ domains, following previous studies [55–57]. Additional file 1: Figure S3 shows the I_D_/I_G_ distribution of Raman test for rHGO thin film. Twenty different locations were tested on the same sample, and I_D_/I_G_ values are located between 1.04 to 1.14.

### Evaluation of Sensing Devices Based on rHGO

**Fig. 5:**
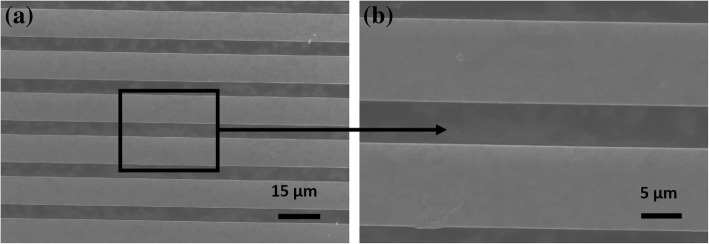
SEM images of **a** rHGO bridged electrode arrays and **b** the enlarged image of selected area

The rHGO thin film was deposited on a silicon substrate according to our previously reported methods [45]. Figure 5 shows the SEM images of rHGO deposited between electrodes. The rHGO sheets were distributed between the two electrodes, forming a good network structure. The resistance response of the resulting sensing device was measured using an accurate semiconductor measuring instrument (Agilent 4156C). The resistance of ~ 1 MΩ at a voltage of 500 mV indicates that a good conductive circuit of the rHGO-based sensor was prepared. Additional file 1: Figure S4 shows the resistance distribution of 50 rHGO thin-film gas sensors.

NH_3_, a toxic gas, is very harmful to human health, which is widely used in various fields such as plastics, fertilizers, and medicine [56]. It is important to study NH_3_ gas sensors for detecting NH_3_ leakage. The response of rHGO sensor was measured with different concentrations of NH_3_ gas. The following formula was used to calculate the concentration of NH_3_ [48]:

1 $$ {F}_{{\mathrm{NH}}_3}=\frac{P_{{\mathrm{NH}}_3}}{P_0-{P}_{{\mathrm{NH}}_3}}{F}_{\mathrm{C}} $$

where *F*_c_ (sccm) is the carrying gas flow, *P*_0_ is the pressure at the outlet of bubbling bottle, and ${P}_{{\mathrm{NH}}_3}$ is the pressure of NH_3_ [58].

2 $$ {C}_{{\mathrm{NH}}_3}\left(\mathrm{ppm}\right)=\frac{10^6{F}_{{\mathrm{NH}}_3}}{F_{\mathrm{d}}+{F}_{\mathrm{C}}+{F}_{{\mathrm{NH}}_3}} $$

where *F*_*d*_ is the flow of compressed air diluted with NH_3_ gas.

The resistance response performance of sensor (R) was calculated using the following formula:

3 $$ R\left(\%\right)=\frac{\Delta R}{R_0}\times 100=\frac{R_{{\mathrm{NH}}_3}-{R}_0}{R_0}\times 100 $$

where *R*_*0*_ and ${R}_{{\mathrm{NH}}_3}$ are the resistance of sensor before and after contacting with NH_3_ gas, respectively.

**Fig. 6:**
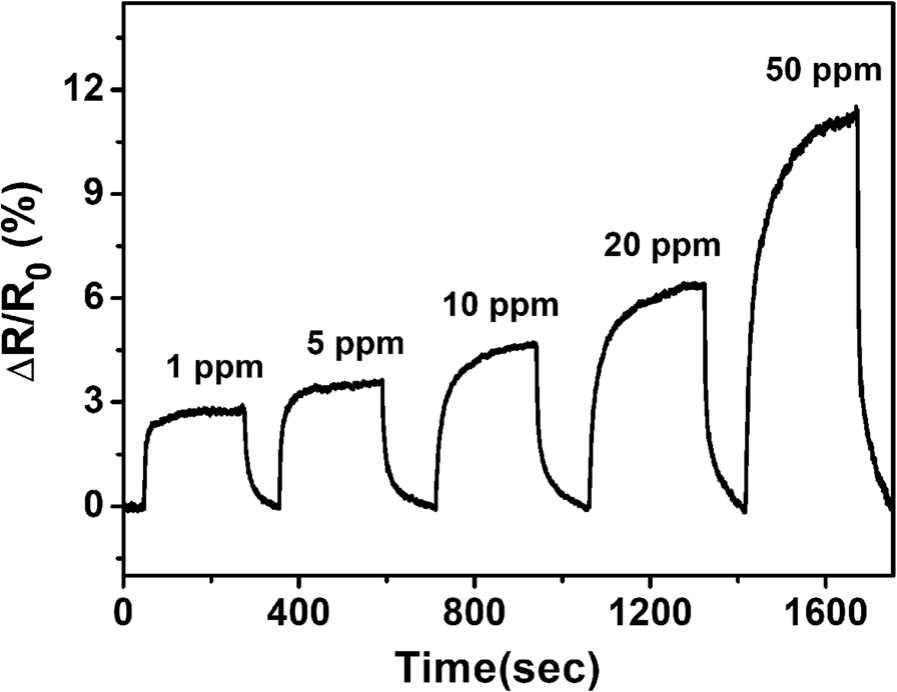
Plot of normalized resistance change versus time for the sensing device based on rHGO upon exposure to NH_3_ with concentrations ranging from 1 to 50 ppm

Figure 6 shows the real-time resistance response of sensing device based on rHGO thin film exposed to various concentrations of NH_3_ (1–50 ppm) and then recovered in dry air at room temperature. The rHGO thin-film gas sensor exhibits good reversible response to different concentrations of NH_3_. When NH_3_ enters the chamber, the resistance of sensor significantly increases within 4 min. An increase in the concentration of NH_3_ results in a corresponding increase in sensor resistance. When the sensor is exposed to NH_3_ at a concentration of 1–50 ppm, the change in resistance is clearly observed. When 50 ppm NH_3_ is passed into the test chamber, the sensor exhibits a resistance change of 11.32%. Even for a sensor with NH_3_ concentration as low as 1 ppm, a resistance responsibility of 2.81% is achieved. The recovery characteristics of rHGO thin-film gas sensor towards different concentrations were calculated as shown in Fig. 6, which can be recovered to 90% of its initial value by flowing dry air without UV/IR light illumination or thermal treatment.

The high sensitivity of rHGO thin-film gas sensor can be attributed to its large specific surface area, high pore volume, and good electrical connection between the rHGO thin film and electrodes. The *p*-type semiconductor characteristics of rHGO thin-film gas sensor can be attributed to the existing oxygen-based moieties and structural defects [59, 60], inducing a hole-like carrier concentration. NH_3_ is a reducing agent with a lone electron pair [61]. When the sensor is exposed to electron-donating NH_3_ molecules, electrons can be easily transferred to *p*-type rHGO thin film, thereby reducing the number of conductive holes in the rHGO valence band. This hole (or *p*-type doping) shifts the Fermi level farther away the valence band, thus increasing the resistance of rHGO sensors. The rHGO thin film prepared by photo-Fenton reaction forms many micropores on the surface of graphene film, and NH_3_ can completely interact with rHGO thin film, so that the sensor device has a high sensitivity and stable working performance. After reduction, PPy molecules were adsorbed on the surface of rHGO. A small amount of PPy molecule adsorption, as a conductive polymer, might play an important role in enhancing the interaction between NH_3_ gas and sp^2^-bond carbon of rHGO [52]. The simple, low-cost sensors with a high sensitivity can be used as an ideal NH_3_ gas detection device and have broad prospects in practical applications.

**Fig. 7:**
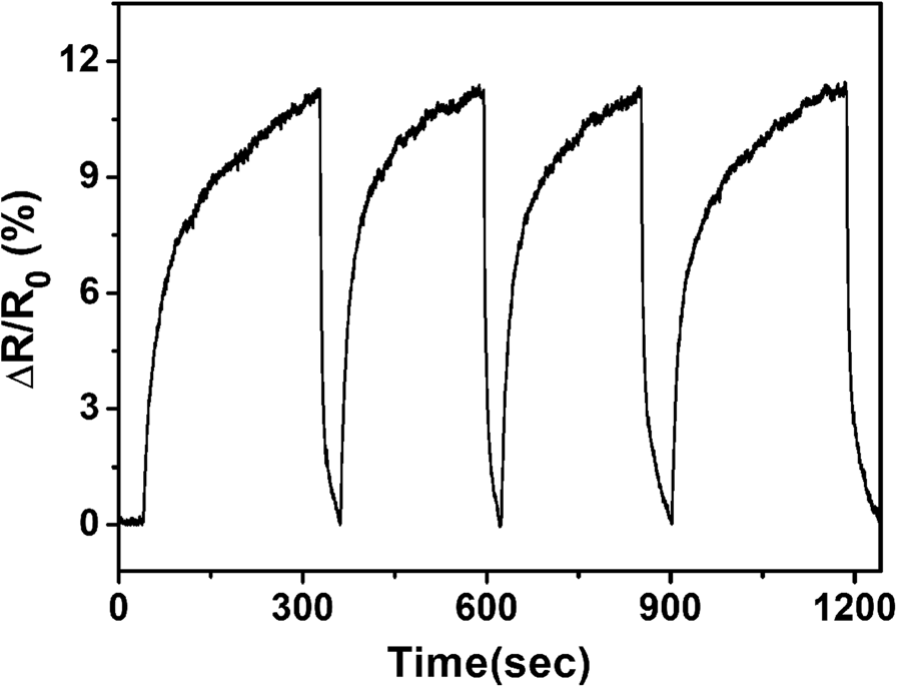
Repeatability of response of rHGO thin-film sensor to 50 ppm NH_3_

For practical testing, sensor repeatability is an important evaluation criterion. The rHGO thin-film sensor was exposed to 50 ppm of NH_3_ for four consecutive cycles. As shown in Fig. 7, the gas sensors based on rHGO exhibits a high reproducibility. After repeated exposure to the gas and recovery cycles, the sensor’s resistance response remained stable, reaching a constant value of 11.32%. When the NH_3_ flow is turned off and background gas was introduced, the resistance of sensor returns to its original value within 2 min. In addition, the performance of rHGO thin-film gas sensor is very stable over several months.

**Fig. 8:**
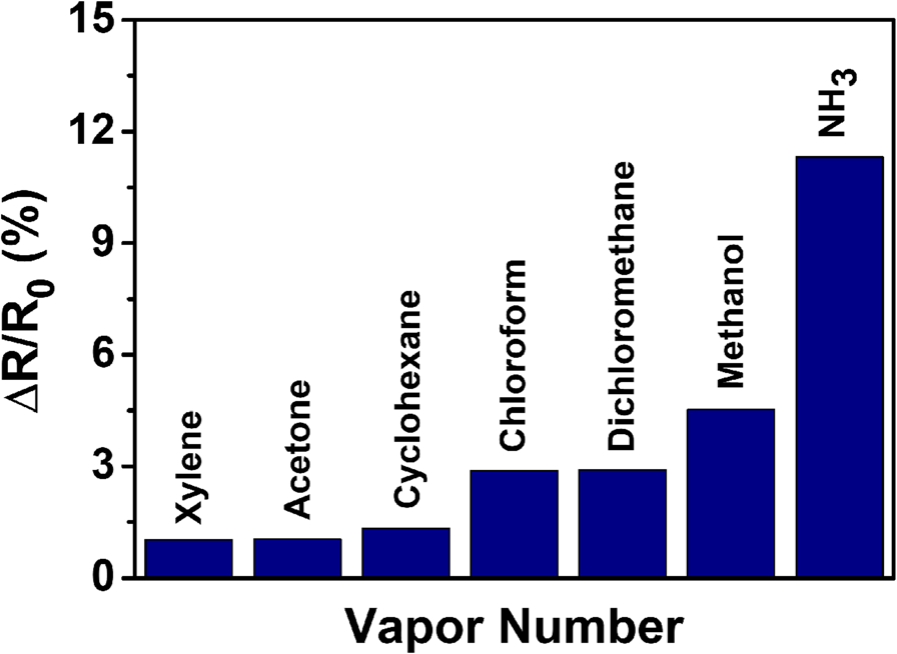
Response of rHGO thin-film gas sensors to NH_3_ compared with other analytes diluted to 1% of saturated vapor concentration

The selectivity of rHGO thin-film gas sensor was evaluated and reported in Fig. 8 for different gases, including xylene, acetone, cyclohexane, chloroform, dichloromethane, and methanol. The saturation concentration of other vapors was generated by bubbling at room temperature and diluted to 1% with dry air. The pressure at the outlet of the bubbler was atmospheric (*P*_*0*_). As shown in Fig. 8, the sensor exhibits excellent selectivity for NH_3_. The response of rHGO thin-film gas sensor to 50 ppm of NH_3_ is 2.5 times more than the response to other analytes. Notably, the concentration of other analytes is much higher than that of NH_3_. These results indicate that rHGO thin-film gas sensor is highly selective and can be considered as an excellent sensing material for the detection of NH_3_.

## Conclusions

In summary, we developed a novel NH_3_ sensor based on holey graphene thin films. HGO nanosheets were prepared by the etching of GO by photo-Fenton reaction. rHGO was formed by the reduction of HGO with pyrrole. rHGO thin-film gas sensors were fabricated by the drop drying of rHGO suspensions on electrodes. The rHGO thin-film gas sensors have excellent NH_3_ sensing properties such as high responsivity, fast response, and short recovery time. Compared with 1% of saturated vapors of other gases, the response of rHGO thin-film gas sensors to ammonia is more than 2.5 times of other interfering gases. Such rHGO thin-film gas sensors indeed pave the path for the next generation of rGO-based sensing devices with dramatically improved performance as well as facile fabrication routes.

## Additional Files

Additional file 1: **Figure S1.** An enlarged AFM image of GO sheets after reaction with Fenton reagent under UV irradiation for 1 h. **Figure S2.** AFM image (a) and height profile (b) of GO sheets before reaction with Fenton reagent. **Figure S3.** The ID/IG distribution of Raman test for rHGO thin-film: 20 different locations were tested on the same sample. **Figure S4.** The resistance distribution of 50 rHGO thin-film gas sensors. (DOC 14183 kb)

## Data Availability

All data generated or analyzed during this study are included in this published article.

## Abbreviations

2D: Two-dimensional
AFM: Atomic force microscope
GO: Graphene oxide
HGO: Holey graphene oxide
NH_3_: Ammonia
PPy: Polypyrrole
rGO: Reduced graphene oxide
rHGO: Reduction holey graphene oxide
SEM: Scanning electron microscopy
XPS: X-ray photoelectron spectroscopy
