# Supplementary material for: Gas Sensors Based on Chemically Reduced Holey Graphene Oxide Thin Films
Source: Nanoscale Res Lett. 2019 Jul 1;14:218. doi: 10.1186/s11671-019-3060-5 (PMC6603111; doi:10.1186/s11671-019-3060-5)
Supplement: Supplementary file 1 — Figure S1. An enlarged AFM image of GO sheets after reaction with Fenton reagent under UV irradiation for 1 h. Figure S2. AFM image (a) and height profile (b) of GO sheets before reaction with Fenton reagent. Figure S3. The ID/IG distribution of Raman test for rHGO thin-film: 20 different locations were tested on the same sample. Figure S4. The resistance distribution of 50 rHGO thin-film gas sensors. (DOC 14183 kb) [file 11671_2019_3060_MOESM1_ESM.doc]

Supplementary Material for

Gas sensors based on chemically reduced holey graphene oxide thin films

**Ming Yang^1,2^, Yanyan Wang^1,2*^, Lei Dong^1,2^, Zhiyong Xu^1,2^, Yanhua Liu^1,2^, Nantao Hu^4^, Eric Siu-Wai Kong^4^, Jiang Zhao^3^, Changsi Peng^1,2*^**

**^1^** School of Optoelectronic Science and Engineering & Collaborative Innovation Center of Suzhou Nano Science and Technology, Soochow University, Suzhou 215006, People's Republic of China

**^2^** Key Lab of Advanced Optical Manufacturing Technologies of Jiangsu Province & Key Lab of Modern Optical Technologies of Education Ministry of China, Soochow University, Suzhou 215006, People's Republic of China

**^3^** Jiangsu Provincial Engineering Laboratory for RF Integration and Micropackaging, College of Electronic and Optical Engineering & College of Microelectronics, Nanjing University of Posts and Telecommunications, Nanjing 210023, People's Republic of China

**^4^** Key Laboratory for Thin Film and Microfabrication of Ministry of Education, Department of Micro/Nano Electronics, School of Electronic Information and Electrical Engineering, Shanghai Jiao Tong University, Shanghai 200240, People's Republic of China

*Corresponding author: [yywang@suda.edu.cn](mailto:yywang@suda.edu.cn); [changsipeng@suda.edu.cn](mailto:changsipeng@suda.edu.cn)


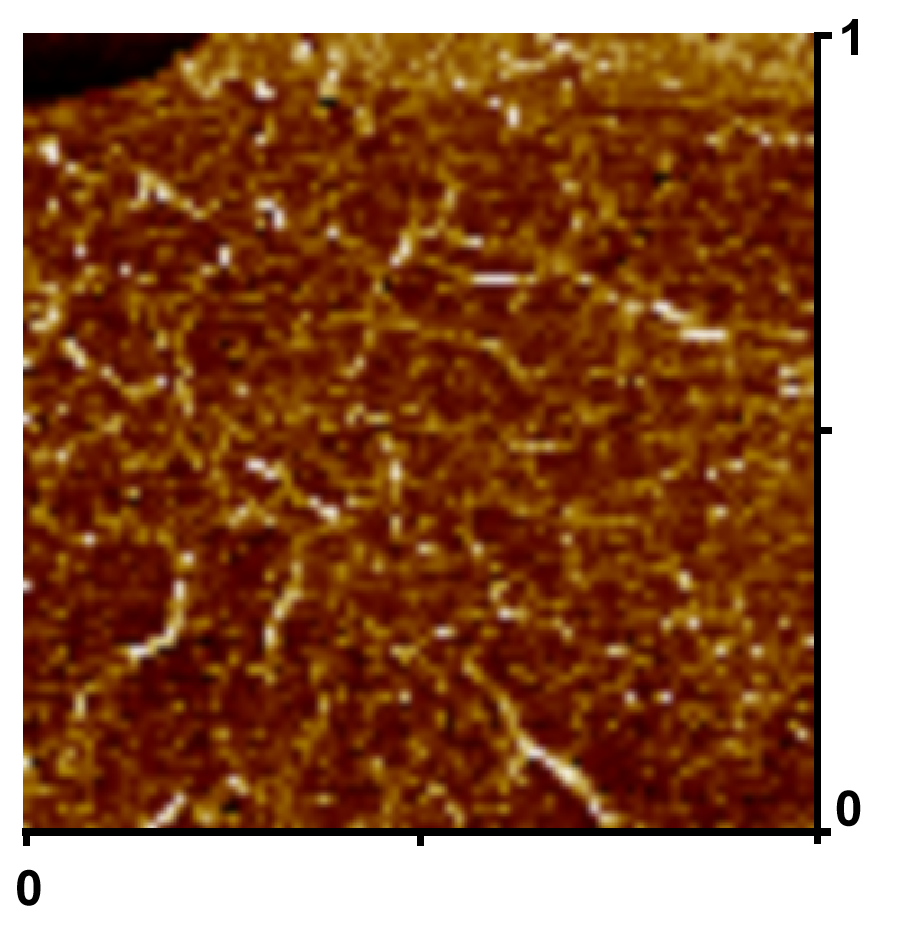


Fig. S1. An enlarged AFM image of GO sheets after reaction with Fenton reagent under UV irradiation for 1 h.


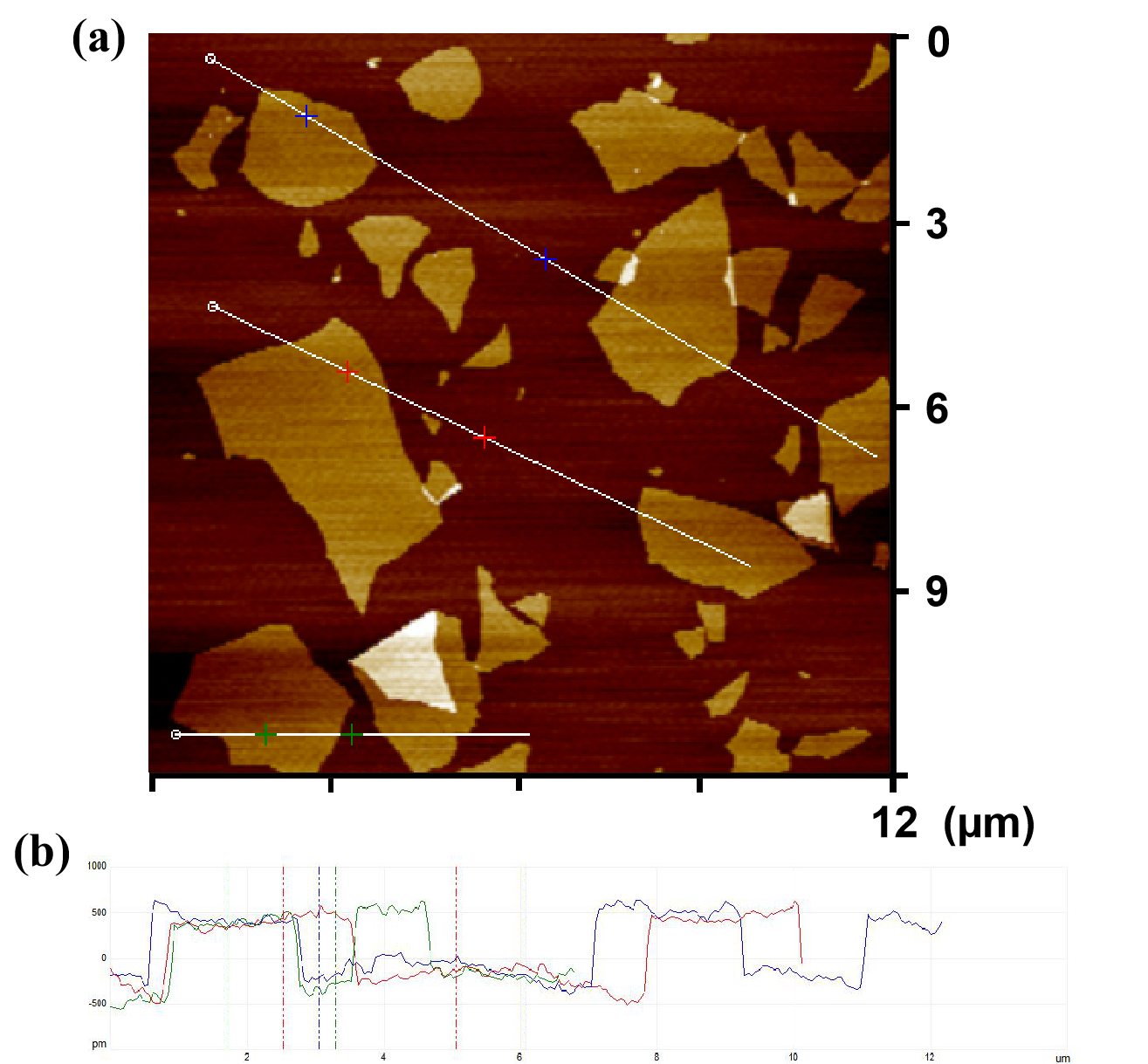


Fig. S2. AFM image (a) and height profile (b) of GO sheets before reaction with Fenton reagent.





Fig. S3. The I_D_/I_G_ distribution of Raman test for rHGO thin-film: 20 different locations were tested on the same sample.





Fig. S4. The resistance distribution of 50 rHGO thin-film gas sensors.
